# 

**Published:** 1912-07

**Authors:** 


					﻿PUBLISHED MONTHLY.
SUBSCRIPTION $1.00
ATLANTA
JOURNAL-RECQR^
OF MEDICINE*;9'1
( Atlanta Medical and Surgical Journal, Established 1»55’. 7c7|L Vflnr
Successor to 5	Medical Record, Established 1870.	) 31 111 I LU I
Vol. LIX.
Atlanta, Ga., July, 1912.
No. 4
				

